# Comparison of three malnutrition risk screening tools in identifying malnutrition according to Global Leadership Initiative on Malnutrition criteria in gastrointestinal cancer

**DOI:** 10.3389/fnut.2022.959038

**Published:** 2022-08-04

**Authors:** Yangyang Huang, Ying Chen, Lu Wei, Yan Hu, Liya Huang

**Affiliations:** Department of Geriatrics, Xinhua Hospital, Shanghai Jiao Tong University School of Medicine, Shanghai, China

**Keywords:** malnutrition, gastrointestinal cancer, nutritional risk, screening tool, diagnostic test

## Abstract

**Background:**

Malnutrition is common in patients with gastrointestinal cancer. The first step in the diagnosis of malnutrition is to evaluate the malnutrition risk by validated screening tools according to the Global Leadership Initiative on Malnutrition (GLIM). This study aimed to determine the best nutritional screening tool for identifying GLIM malnutrition and validate the performance of these tools in different age subgroups.

**Materials and methods:**

We did a prospective cohort study of patients who were diagnosed with gastrointestinal cancer from February 2016 to November 2019. The sensitivity, specificity, positive predictive values (PPV), negative predictive values (NPV), and area under the receiver operating characteristic (ROC) curve (AUC) of three screening tools (Nutritional risk screening 2002 (NRS 2002), Geriatric Nutritional Risk Index (GNRI), MNA-SF) were calculated.

**Results:**

A total of 488 patients were enrolled, and 138 patients (28.27%) were malnutrition according to the GLIM criteria. The consistency of NRS 2002, GNRI, and MNA-SF with GLIM-defined malnutrition was 74.8, 72.1, and 71.1%, respectively. In the subgroup analysis of young patients (<65 years), NRS 2002 exhibited the best discrimination with the AUC of 0.724 (95% CI, 0.567–0.882), the sensitivity of 64.3% (95% CI, 35.6–86.0), and the specificity of 80.6% (95% CI, 69.2–88.6). In patients older than 65 years, MNA-SF exhibited the best discrimination with the AUC of 0.764 (95% CI, 0.714–0.814), the sensitivity of 82.3% (95% CI, 74.1–88.3), and the specificity of 70.5% (95% CI, 64.7–75.7).

**Conclusions:**

Nutritional risk screening 2002 (NRS 2002) is the best malnutrition screening tool in gastrointestinal cancer patients younger than 65 years, and MNA-SF is the best malnutrition screening tool in patients older than 65 years. It is necessary to select targeted nutritional screening tools according to the difference in age.

## Introduction

Malnutrition is common in geriatric gastrointestinal cancer patients. Many studies have found that malnutrition is associated with a high frequency of complications and mortality in patients with gastrointestinal cancer (1, 2). Thus, it is necessary to perform nutritional interventions to reduce complications and mortality of gastrointestinal cancer patients.

The diagnostic criteria for malnutrition are constantly being updated and improved. A newly published malnutrition criterion of The Global Leadership Initiative on Malnutrition (GLIM) has gathered many global clinical nutrition societies and incorporated multiple common malnutrition criteria which aimed to standardize the malnutrition diagnosis. GLIM criteria for diagnosis of malnutrition consist of two steps risk screening and malnutrition diagnosis. The key first step is to use any validated malnutrition screening tool to identify “at risk” status, to choose the best malnutrition screening tool that can improve the efficiency of malnutrition detection and avoid the waste of human resources (3). However, it is unclear which screening tools can better screen for malnutrition in patients with gastrointestinal cancer.

Nutritional risk screening 2002 (NRS 2002), the Geriatric Nutritional Risk Index (GNRI), and mini nutritional assessment short form (MNA-SF) are commonly used nutritional screening scales. In terms of specific evaluation indicators, NRS 2002 includes three parts disease severity, nutritional impairment and age, GNRI based on serum albumin level and body weight, and MNA-SF consists of 6 questions on food intake, weight loss, mobility, psychological stress or acute disease, presence of dementia or depression, and body mass index (BMI).

The aim of our study was to compare the three common malnutrition risk screening tools (NRS 2002, GNRI, and MNA-SF), aiming to find the best screening tool for identifying GLIM malnutrition and to validate their performance in different age subgroups, which helps to improve the efficiency of malnutrition detection and avoid the waste of medical resources.

## Patients and methods

### Patients

The study included patients who underwent curative surgery for gastrointestinal cancer from February 2016 to November 2019 in two centers. Inclusion criteria included: (1) Patients who underwent elective curative surgery for gastrointestinal cancer; (2) Patients who signed informed consent and agreed to participate in this study. The exclusion criteria were: (1) Patients who have undergone a palliative or emergency operation; (2) Patients who had difficulty in data collection during NRS 2002, GNRI, and MNA-SF surveys; and (3) Patients who refused to take part in this study.

### Assessment of nutritional risk

Three scales were collected within 24 h of admission aiming to record the baseline nutritional risk status and are carried out by a trained specialist. NRS 2002, GNRI, and MNA-SF were used as the tools to screen malnutrition risk by clinical investigators. For NRS 2002 scale, patients with a total score ≥ 3 were considered at nutritional risk (4). The GNRI values were divided into 4 categories as major risk (GNRI: <82), moderate risk (GNRI: 82 to <92), low risk (GNRI: 92 to ≤ 98), and no risk (GNRI: >98) (5). The MNA-SF scores were converted into two categories as normal nutritional status (12–14 points) and at risk of malnutrition (≤ 11 points) (6).

### Diagnosis of malnutrition

Malnutrition was diagnosed according to GLIM criteria. A two-step approach for the malnutrition diagnosis was selected, first screening to identify “at risk” status by the use of any validated screening tool, and second, assessment for diagnosis and grading of the severity of malnutrition. Patients were diagnosed as malnourished if any of three phenotypic criteria (non-volitional weight loss, low body mass index, and reduced muscle mass) and either of two etiological criteria (reduced food intake or assimilation, and disease burden/inflammation) were met. Non-volitional weight loss was defined as > 5% within the past 6 months or > 10% beyond 6 months. We assessed low body mass index using Asian BMI data (< 18.5 if < 70 years, or < 20 if > 70 years) (3). Reduced muscle mass was defined using CT-derived skeletal muscle index (SMI) cutoffs (7). Reduced food intake was assessed if food intake was poor (the reduction ratio ≥ 1/4) for a week or more before being admitted to the hospital. In regards to inflammation, chronic inflammation significantly contributes to oncogenesis (8). Hence, all patients with gastrointestinal cancer satisfied the criteria for inflammation/disease burden.

### Statistical analysis

Continuous variables with normal distribution were presented as mean ± standard deviation (SD) and analyzed using a *t*-test. Continuous variables with non-normal distribution were presented as the median and interquartile range (IQR) and analyzed using Mann–Whitney U test, and categorical variables were presented as frequencies and analyzed using Pearson’s χ*2* test (or Fisher’s exact test). Univariate logistic analysis was performed for potential baseline predictors and the odds ratios (ORs) with their 95% CIs were calculated. Variables with a trend (*p* < 0.10) in the univariate analysis were selected as potential parameters, and then, a forward stepwise variable selection was used to establish a multivariate logistic regression. Receiver operating characteristic (ROC) curves were conducted to evaluate the performance of the NRS 2002, GNRI, and MNA-SF in identifying malnutrition. The area under the curve (AUC), sensitivity, specificity, positive predictive value (PPV), negative predictive value (NPV), positive likelihood ratio, and negative likelihood ratio were also calculated. The *p*-value of < 0.05 was considered statistically significant. All data were analyzed using SPSS software Version.23.0 (IBM, Armonk, NY, United States).

## Results

A total of 488 geriatric gastrointestinal cancer patients enrolled in the study, there were 317 (65%) males and 171 (35%) females. For cancer types, there were 151 (30.9%) gastric cancer and 337 (69.1%) colorectal cancer. A total of 138 patients (28.27%) were diagnosed with malnutrition according to GLIM criteria. The baseline characteristics between malnutrition and non-malnutrition groups are shown in Table 1. The average age of patients in the malnutrition group was 74 (69–80), while the non-malnutrition group had a significantly lower age of 71 (65.75–77.25). The weight, BMI, handgrip strength, gait speed, hemoglobin, serum albumin, and lymphocyte count were significantly lower in the malnutrition group than in the non-malnutrition group. Additionally, the neutrophil counts and C-reaction protein (CPR) were significantly higher in the malnutrition group than in the non-malnutrition group.

**TABLE 1:** Baseline characteristics between malnutrition and non-malnutrition groups.

Factors	Malnutrition (*n* = 138)	Non-malnutrition (*n* = 350)	*P*-value
Age, year	74 (69–80)	71 (65.75–77.25)	0.001*
Sex, male	91 (65.9%)	226 (64.6%)	0.775
Height, cm	164 (157–170)	165 (159–170)	0.190
Weight, kg, mean ± SD	58.24 ± 10.38	64.88 ± 11.18	< 0.001*
BMI, kg/m^2^, mean ± SD	21.78 ± 3.08	24.03 ± 3.52	< 0.001*
Hypertension, yes	60 (43.5%)	160 (45.7%)	0.655
Diabetes, yes	29 (21.0%)	68 (19.4%)	0.693
Handgrip strength, kg	23.70 (18.35–31.35)	27.45 (19.73–34.80)	0.002*
Gait speed, m/s	0.91 (0.71–1.06)	1.01 (0.85–1.16)	< 0.001*
Hemoglobin, g/L	106 (84.75–121.25)	125 (110–138)	< 0.001*
Serum albumin, g/L	38 (34–41)	43 (39–45)	< 0.001*
Neutrophil counts, 10^9^/L	3.96 (2.98–5.33)	3.50 (2.68–4.45)	< 0.001*
Lymphocyte count, 10^9^/L	1.44 (1.10–1.75)	1.60 (1.23–1.99)	0.001*
CPR, mg/L	9.37 (3.17–23.48)	3.23 (3.02–3.59)	< 0.001*
Cancer types			0.473
Gastric cancer	46 (33.3%)	105 (30.0%)	
Colorectal cancer	92 (66.7%)	245 (70.0%)	
Combined resection, yes	6 (4.3%)	9 (2.6%)	0.306
TNM stages			0.030*
0	2 (1.4%)	16 (4.6%)	
I	17 (12.3%)	77 (22.0%)	
II	47 (34.1%)	111 (31.7%)	
III	69 (50.0%)	136 (38.9%)	
IV	3 (2.2%)	10 (2.9%)	
Operative duration, min	143.5 (123.75–170.25)	152 (120–182)	0.243

Data is represented as median (25th–75th) or number (%), unless otherwise stated.

BMI, Body mass index; CPR, C-reaction protein; TNM, tumor-node-metastasis.

*Statistically significant.

**FIGURE 1:**
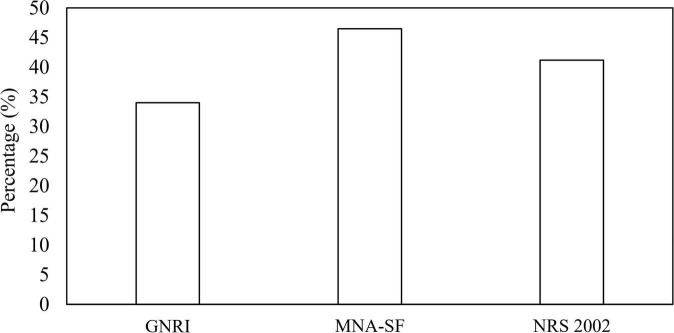
Classification of malnutrition risk based on GNRI, MNA-SF, and NRS 2002. GNRI, geriatric nutritional risk index; MNA-SF, mini nutritional assessment-short form; NRS, nutritional risk screening.

The prevalence of malnutrition risk based on GNRI, MNA-SF, and NRS 2002 was shown in Figure 1. The proportions of being at risk of malnutrition were 34, 46.5, and 41.2% according to GNRI, MNA-SF, and NRS 2002, respectively. Table 2 shows the consistency between GNRI, MNA-SF, and NRS 2002 with GLIM-malnutrition. The consistency of NRS 2002, GNRI, and MNA-SF with GLIM-defined malnutrition was 74.8, 72.1, and 71.1%, respectively. **Supplementary Table 1** lists the results of univariate and multivariate analyses for predictors of GLIM malnutrition. Baseline characteristics with *p* < 0.01 were included in the multivariate analyses with NRS 2002, GNRI, and MNA-SF, respectively. In the model with NRS 2002, we found that the NRS 2002, BMI, and serum were independent factors of malnutrition; in the model with MNA-SF, the MNA-SF, BMI, serum, and hemoglobin were independent factors of malnutrition; however, in the model with GNRI, GNRI were excluded in the multivariate analysis.

**TABLE 2:** Consistency between GNRI, MNA-SF, and NRS 2002 with GLIM-malnutrition.

Factors	Malnutrition (*n* = 138)*^a^*	Non-malnutrition (*n* = 350)
**GNRI**		
At risk (score ≤ 98)	84 (60.9%)	82 (23.4%)
No risk (score > 98)	54 (39.1%)	268 (76.6%)
**MNA-SF**		
At risk (score ≤ 11)	112 (81.2%)	115 (32.9%)
No risk (score > 11)	26 (18.8%)	235 (67.1%)
**NRS 2002**		
At risk (score ≥ 3)	108 (78.3%)	93 (26.6%)
No risk (score < 3)	30 (21.7%)	257 (73.4%)

GNRI, geriatric nutritional risk index; MNA-SF, mini nutritional assessment-short form; NRS, nutritional risk screening.

^a^Malnutrition is defined according to the GLIM criteria.

**FIGURE 2:**
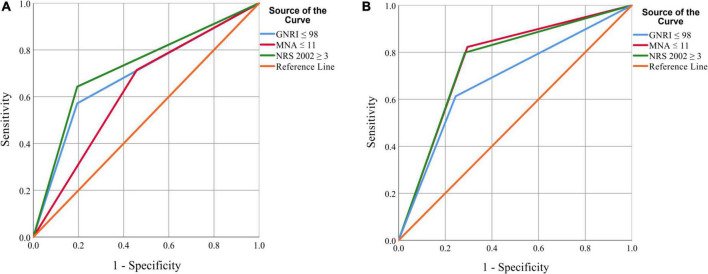
ROC curves of the three nutritional screening scales in diagnosing malnutrition in patients younger **(A)** or older than 65 years **(B)**. The area under the curve (AUC) is 0.688, 0.628, 0.724 for GNRI, MNA-SF, and NRS 2002 in patients younger than 65 years, and is 0.684, 0.764, 0.757 for GNRI, MNA-SF, and NRS 2002 in patients aged 65 years or older.

To examine the discriminative ability of screening tools, the ROC curves of NRS 2002, GNRI, and MNA-SF were plotted using GLIM-defined malnutrition as a control in Figure 2. For patients younger than 65 years old, NRS 2002 exhibited the best discrimination with an AUC of 0.724 (95% CI, 0.567–0.882), and the AUC for MNA-SF and GNRI was 0.628 (95% CI, 0.472–0.784) and 0.688 (95% CI, 0.524–0.853), respectively. For patients older than 65 years old, MNA-SF exhibited the best discrimination with an AUC of 0.764 (95% CI, 0.714–0.814), and the AUC for NRS 2002 and GNRI were 0.757 (95% CI, 0.706–0.808) and 0.684 (95% CI, 0.626–0.742), respectively.

The sensitivity, specificity, PPV, and NPV were also calculated in Tables 3, 4. For patients younger than 65 years old, the Youden index (sensitivity + specificity-1) was 0.449, 0.256, and 0.377 for NRS 2002, GNRI, and MNA-SF, respectively. For patients older than 65 years old, the Youden index was 0.514, 0.368, and 0.528 for NRS 2002, GNRI, and MNA-SF, respectively.

**TABLE 3:** Performance of three nutritional screening scales in the diagnosis of malnutrition in patients younger than 65 years old.

	GNRI	MNA-SF	NRS 2002
Sensitivity (%)	57.1 (29.6–81.2)	71.4 (42.0–90.4)	64.3 (35.6–86.0)
Specificity (%)	80.6 (69.2–88.6)	54.2 (42.1–65.8)	80.6 (69.2–88.6)
Positive predictive value,%	36.4 (18.0–59.2)	23.3 (12.3–39.0)	39.1 (20.5–61.2)
Negative predictive value,%	90.6 (80.1–96.1)	90.7 (76.9–97.0)	92.1 (81.7–97.0)
Positive likelihood ratio	2.94 (1.53–5.65)	1.56 (1.03–2.36)	3.31 (1.79–6.09)
Negative likelihood ratio	0.53 (0.29-0.98)	0.53 (0.23–1.24)	0.44 (0.22–0.90)

Values are given as percentages (95% CI) or ratios (95% CI).

GNRI, geriatric nutritional risk index; MNA-SF, mini nutritional assessment-short form; NRS, nutritional risk screening.

**TABLE 4:** Performance of three nutritional screening scales in the diagnosis of malnutrition in patients 65 years of age or older.

	GNRI	MNA-SF	NRS 2002
Sensitivity (%)	61.3 (52.1–69.8)	82.3 (74.1–88.3)	79.8 (71.5–86.3)
Specificity (%)	75.5 (70.0–80.4)	70.5 (64.7–75.7)	71.6 (65.8–76.7)
Positive predictive value,%	52.8 (44.3–61.1)	55.4 (47.9–62.7)	55.6 (48.0–63.0)
Negative predictive value,%	81.4 (76.0–85.8)	89.9 (84.9–93.4)	88.8 (83.8–92.5)
Positive likelihood ratio	2.51 (1.95–3.22)	2.79 (2.28–3.40)	2.81 (2.29–3.45)
Negative likelihood ratio	0.51 (0.41–0.64)	0.25 (0.17–0.37)	0.28 (0.20–0.40)

Values are given as percentages (95% CI) or ratios (95% CI).

GNRI, geriatric nutritional risk index; MNA-SF, mini nutritional assessment-short form; NRS, nutritional risk screening.

## Discussion

The GLIM is a consensus report from the global clinical nutrition community for the diagnosis of malnutrition. Since the first key step is to identify “at risk” patients, nutritional screening tools should be effective and easy to perform. Although malnutrition is a frequent problem in patients with gastrointestinal cancer, few studies investigated the efficiency of screening tools in identifying GLIM-defined malnutrition. As far as we know, this is the first study investigating the efficiency of the malnutrition risk tool for identifying GLIM-defined malnutrition in the gastrointestinal cancer population of both sexes.

Nutritional risk varied greatly, ranging from 40 to 90%, depending on the nutritional screening tool used (9). Meanwhile, there are differences and similarities between various malnutrition screening tools, but the results of still contradictory. Myoungha et al. evaluated five nutritional screening tools and showed that MNA-SF overestimated nutritional risk while NRS 2002 performed better than MNA-SF in the elderly (10). In contrast, Poulia’s study demonstrated that NRS 2002 overestimated the nutritional risk while MNA-SF had better validity and must have the best validity and the greatest consistency in the assessment of malnutrition in the elderly (9). Understandably, these studies come to different conclusions, probably because of different definitions of what exactly counts as malnutrition.

According to previous studies, the prevalence of malnutrition in the gastrointestinal is about 30–50% (11–13). According to GLIM criteria, the prevalence of malnutrition was 28.27% in our study which is also consistent with previous studies (14–16). For the reason why our study population had a relatively lower incidence of malnutrition, we hypothesized that it might be due to the higher incidence of colorectal cancer and the almost lack of stage IV cancer patients in our study. In our study, we aimed to find the highest efficient malnutrition screening tool in the first step of the GLIM diagnosis.

The higher the sensitivity of the malnutrition screening scale, the fewer patients diagnosed malnourished by GLIM were missed by the nutrition screening scale. The sensitivity is the proportion of the positive samples that were correctly classified, and the specificity represents the proportion of the negative samples that were correctly classified. A higher sensitivity helps us to identify more patients at risk of malnutrition, while a higher specificity helps to save medical resources. In our study, in patients younger than 65 years, MNA-SF had the highest sensitivity (71.4%) but lowest specificity (54.2%), while GNRI and NRS 2002 had the highest specificity (80.6%). Additionally, NRS 2002 had the greatest AUC compared with GNRI and MNA-SF. Although MNA-SF had the highest sensitivity, NRS 2002 had the highest sensitivity and the greatest AUC, therefore, we considered NRS 2002 prior to GNRI and MNA-SF as the malnutrition screening tool in gastrointestinal cancer patients younger than 65 years. In patients older than 65 years, MNA-SF had the highest sensitivity (82.3%), GNRI had the highest specificity (75.5%), and MNA-SF had the greatest AUC compared with GNRI and NRS 2002. Thus, we considered that MNA-SF is prior to NRS 2002 and GNRI as the malnutrition screening tool in identifying malnutrition risk in geriatric patients with gastrointestinal cancer.

The varying results of this comparison can be attributed to differences in the original design of the three screening tools. The NRS 2002 was designed to identify patients at increased nutritional risk expected to benefit from nutritional support (4). NRS 2002 shows validity to screen for malnutrition among different hospitalized populations and age groups in many studies (17, 18). The original design of the MNA-SF was also the first step toward full nutritional assessment (6, 19), and MNA-SF showed excellent sensitivity to either reference method, but poor specificity (20), which classifies too many patients at risk of malnutrition compared to other reference methods. Elderly patients are more likely to be potentially malnourished due to the gradual aging of their organs, so it is acceptable to include too many patients as those at risk of malnutrition. Other studies have also confirmed the effectiveness of MNA-SF screening for malnutrition in elderly patients. Poulia et al. evaluated the efficacy of six nutritional screening tools in the elderly, MNA-SF was proven to have great validity (9). Yoshinari Matsumoto et al reported that, when using the MNA-SF as the screening tool of GLIM, they identified malnourished patients with high accuracy (21). The GNRI has been designed specifically for the elderly with the purpose to predict clinical outcomes (morbidity, mortality, (postoperative) complications, or length of hospital stay), which consists of albumin, weight, and ideal weight (5).

We can see both NRS 2002 and MNA-SF have several similar diagnostic criteria to GLIM. In NRS 2002, three criteria (weight loss, BMI, and food intake) were similar to GLIM, and in MNA-SF, four criteria (weight loss, BMI, change of appetite, disease burden) were similar to GLIM. These criteria were feasible and easy to operate, but only reduced muscle mass which only GLIM had was labor-intensive. Therefore, it is practical to use NRS 2002 and MNA-SF as screening tools for gastrointestinal cancer patients.

This study has some limitations. Firstly, the screening tools can be used only if the patient is able to communicate or if a family member is able to answer the interviewer’s questions. This could bias the study by excluding some malnourished elderly patients who could not participate. Secondly, this study was obviously limited by the inclusion of only patients with curable gastric cancer.

## Conclusion

The first step of GLIM criteria requires identifying “at risk” patients by the use of any validated screening tool. In our study, we demonstrated that NRS 2002 was the best malnutrition screening tool in gastrointestinal cancer patients younger than 65 years, and MNA-SF was the best malnutrition screening tool in patients older than 65 years. Therefore, choosing a suitable malnutrition screening tool for different ages helps to detect more malnutrition and contributes to the early detection and treatment of malnutrition.

## Data availability statement

The original contributions presented in this study are included in the article/supplementary material, further inquiries can be directed to the corresponding author.

## Ethics statement

The studies involving human participants were reviewed and approved by Ethics Committees of Shanghai Tenth People’s Hospital and the First Affiliated Hospital of Wenzhou Medical University. The patients/participants provided their written informed consent to participate in this study.

## Author contributions

LH planned the work. YH, LW, and YYH carried out the statistical analysis. YYH and YC wrote and reported the work. LH played a critical role in revising the manuscript. All authors critically assessed and reviewed the manuscript, approved the version to be published, and made a significant contribution to this article.
